# Phenotypic profile and predictors of adverse outcomes during paediatric cardiac catheterisation at an academic hospital, South Africa

**DOI:** 10.3389/fped.2026.1843252

**Published:** 2026-06-25

**Authors:** Crystal Chalwe, Antoinette Cilliers, Palesa Motshabi Chakane, Palesa Mogane

**Affiliations:** 1 Department of Anesthesiology, School of Clinical Medicine, University of the Witwatersrand, Johannesburg, South Africa; 2 Division of Cardiology, School of Clinical Medicine, University of the Witwatersrand, Johannesburg, South Africa

**Keywords:** adverse outcomes, cardiac catheterisation, complications, congenital heart disease, crisp score, paediatrics

## Abstract

**Background:**

Paediatric cardiac catheterization is pivotal in the diagnosis and management of patients with congenital heart disease. Although cardiac referral centres exist, access to cardiac surgery remains limited in low-income countries. The study aimed to describe the demographic and clinical profile, determine adverse outcomes, and identify predictors of complications among children undergoing cardiac catheterization at a state hospital in Johannesburg, Gauteng.

**Methods:**

A retrospective cohort study was performed on children aged 0–18 years with congenital heart disease who presented for cardiac catheterisation between January 2018 and December 2022. Patient data was obtained from the cardiac catheterisation laboratory, anaesthetic records and the paediatric cardiac database. Descriptive and multivariate regression data analysis were performed.

**Results:**

A total of 597 paediatric cardiac catheterization procedures were analysed. The median (IQR) age was 1.8 (0.8–4.0) years with 52% males. Two-thirds, 397 (66%), were on anti-failure treatment and 236 (40%) had pulmonary hypertension. The Catheterisation RISk score for Paediatrics (CRISP) score was significantly associated with complications AOR 1.21 (95% CI 1.10–1.33; *p* < 0.001). Age <1 year was predictive of complications AOR 1.83 (95% CI 1.11–3.01; *p* = 0.018). The presence of a congenital syndrome was also associated with increased odds of complications AOR 1.72 (95% CI 1.10–2.67; *p* = 0.016). Peri-operative administration of inotropes AOR 18.3 (95% CI 6.05–70.0; *p* < 0.001), beta blockers AOR 2.43 (95% CI 1.28–4.54; *p* = 0.006), and prostin AOR 0.25 (95% CI 0.07–0.80; *p* = 0.025) were associated with complications. Mortality in this cohort was 5 (0.8%). Pulmonary hypertension was not associated with increased perioperative complications.

**Conclusion:**

The current study demonstrates that paediatric cardiac catheterisation procedures in a high-burden, resource-limited South African setting are associated with a diverse patient population, including a significant proportion with pulmonary hypertension and those receiving anti-failure therapy. Despite these challenges, the overall incidence of severe adverse outcomes including mortality remained low, supporting the feasibility and safety of these interventions when managed by an experienced multidisciplinary team.

## Introduction

Congenital heart disease (CHD) accounts for nearly one-third of all major birth defects (1, 2) and is a leading contributor to global infant mortality after communicable disease and malnutrition (3). The global prevalence of CHD at birth is estimated at approximately 1.8 cases per 100 live births, with regional variations; estimates from Sub-Saharan Africa range between 2.0 and 2.6 cases per 100 live births (1). Reliable population-based prevalence data for CHD in Africa remain scarce. A systematic review and meta-analysis demonstrated substantial heterogeneity in reported prevalence, which was attributed to gaps in birth registries, limited access to diagnostic services in rural areas and a predominance of data derived from tertiary academic centres in urban areas (4). The Global Burden of Disease study (1990–2017) (1), demonstrated a greater than 50% reduction in CHD-related mortality in high-income countries (HICs), while mortality rates in low- and middle-income countries (LMICs) remained largely unchanged. This would hold true for South Africa as an upper-middle income country (5). Contributing factors include higher birth rates and prevalence of CHD in LMICs, lack of access to healthcare due to underfunded paediatric and congenital heart disease (PCHD) services (6–8), limited infrastructure, and a shortage of skilled health care providers (5, 9, 10).

Optimal Paediatric and Congenital Heart Disease (PCHD) care requires a continuum-of-care model encompassing early detection, referral to appropriate level hospital with cardiology services for confirmatory diagnostics and initiation of pharmacotherapy, cardiac catheterisation (CC) and or referral for cardiac surgery (3, 11). This tiered system depends on coordinated access to specialised resources, including echocardiography, paediatric cardiac anaesthesia, interventional cardiology, paediatric cardiothoracic surgery, perfusion services, intensive care, and specialised nursing (11). Workforce disparities are stark, with most of Sub-Saharan African countries having one cardiothoracic surgeon per 4 million people, where South Africa was the exception with 1.04 cardiothoracic surgeons per 1 million people (12). HICs in comparison had 27–28 surgeons per 1 million people (9). Even in regions with established surgical programmes, available capacity often remains insufficient due to competing demands from acquired and lifestyle associated heart disease (5).

Although cardiac catheterisation is increasingly used in LMICs, outcome data from these contexts are limited; most evidence comes from high-income countries and may not apply to resource-constrained settings. This study therefore aimed to examine the profile and outcomes of paediatric CHD patients undergoing cardiac catheterisation at a major Gauteng referral site. The main objective of the study was to describe the demographic and clinical profile, determine the adverse outcomes, and identify the predictors of complications among this cohort.

## Methodology

### Study design

This was a retrospective cohort study conducted at a tertiary academic hospital in South Africa. Consecutive convenience sampling was utilised, where inclusion criteria were paediatric patients (0–18 years) with congenital heart disease, undergoing cardiac catheterisation between 1 January 2018 and 31 December 2022. Participants with missing records and those that did not meet inclusion criteria, were excluded. Ethics approval was obtained from the Human Research Committee (M230320) of the University of Witwatersrand and all relevant institutional bodies.

### Data collection and management

Study participants were identified from the cardiac catheterisation laboratory register. Clinical and procedural data were extracted from anaesthetic records, cardiac catheterisation reports and the paediatric cardiology database. Missing reports were collected from patients’ files in the paediatric cardiology clinic. Variables collected included demographic, cardiac diagnosis, syndromic associations, comorbidities, medications, procedural characteristics, American Society of Anaesthesia (ASA) classification, anaesthetic technique, procedural urgency, duration and documented perioperative complications (13). Complications were classified into 3 categories cardiac catheterisation-related (CRC), anaesthetic-related (ARC), and patient-related (PRC) complications. CRCs included complications related to vascular access, injury to vascular or cardiac structures, and haemodynamic disturbances such as arrhythmias or hypotension resulting from device placement or balloon inflation. ARCs comprised airway complications (e.g., laryngospasm and difficult intubation), ventilation-related events due to endotracheal tube malposition or underlying pulmonary disease, and haemodynamic instability associated with anaesthetic induction. PRCs included complications attributable to the underlying cardiac condition, pulmonary pathology, or comorbid disease, rather than the catheterisation procedure or anaesthetic management. The severity of complications was categorised as per The report from The International Society of Nomenclature of Paediatric and Congenital Heart Disease: Part 2 - Nomenclature of the complications associated with interventional cardiology (13, 14). The Catheterisation RISk score for Paediatrics (CRISP) was calculated for each patient using the CRISP calculator (Supplementary Table S1) and CRISP supplementary info (Supplementary Table S2) (15). Data were captured using Microsoft Excel spreadsheet before analysis.

### Analysis

The data were analysed using descriptive and inferential statistics. All continuous variables were summarised as medians with interquartile ranges (IQRs) or means with standard deviations (SD) depending on distribution, and categorical variables were reported as counts and proportions with 95% confidence intervals (CI). The Chi-square test or Fisher's exact tests were used to describe the association between the composite of adverse outcomes and the baseline characteristics and CRISP scores for categorical variables, and the Wilcoxon rank-sum tests for continuous variables. General logistic regression models were fitted to estimate the association between each factor and an adverse event, described as an Odds Ratio (OR). Variables with a *p*-value of <0.1 from univariable regression analysis were chosen and used in a forwards and backwards stepwise selection model using the Akaike Information Criterion (AIC) to guide variable selection to choose between similar variables (e.g., Age and Age under 1 year) to avoid multicollinearity. A *p*-value of less than 0.05 indicated significance. Analyses were performed in R version 4.5.2 (R Foundation for Statistical Computing, Vienna, Austria).

## Results

A total of 597 paediatric cardiac catheterization procedures were analysed (Supplementary Figure S1). The median (IQR) age was 1.8 (0.8–4.0) years with a male proportion of 313 (52%) (Table 1). The median (IQR) weight was 10 (7–15) kg, and the median (IQR) haemoglobin 12.5 (11.3–13.9) g/dL. Majority of the procedures were elective 578 (97%), and diagnostic 380 (64%). The most common interventional procedure was patent ductus arteriosus (PDA) device closure 137 (23%). Syndromic diagnoses were present in 179 (29.9%). Most patients had no prior corrective or palliative cardiac surgery 493 (83%). Two-thirds, 397 (66%), were on anti-failure treatment and 236 (40%) had pulmonary hypertension. Most patients had acyanotic cardiac defects 307 (51.4%). The most common cyanotic cardiac defect was Tetralogy of Fallot (TOF) 53 (8.9%) (Supplementary Table S3). The mean (SD) CRISP score of patients at presentation was 4.13 (2.84).

**Table 1:** Demographic and clinical characteristics of the population.

Characteristic *N* = 597		n (%)/Mean (SD)/Median (IQR)
Age (years), Median (IQR)		1.8 (0.8–4.0)
Gender, *n* (%)	Female	284 (48)
	Male	313 (52)
Weight (kg), Median (IQR)		10 (7–15)
Haemoglobin (g/dL), Median (IQR)		12.5 (11.3–13.9)
Procedure Category, *n* (%)	Diagnostic	380 (64)
	Interventional	217 (36)
Interventional Procedure Type, *n* (%)	PDA closure	139 (23)
	Balloon valvuloplasty of PV	18 (3.0)
	VSD closure	10 (1.7)
	Other	50 (8.4)
Elective vs. Emergency, *n* (%)	Elective	578 (97)
	Emergency	19 (3.2)
ASA, *n* (%)	I	0 (0)
	II	102 (17)
	III	452 (76)
	IV	41 (6.9)
	V	2 (0.3)
Any Syndrome, *n* (%)		179 (29.9)
Syndrome, *n* (%)	Down syndrome	114 (64)
	Di George syndrome	49 (27)
	Noonan syndrome	5 (2.8)
	William syndrome	11 (6.1)
Any Surgery, *n* (%)		104 (17)
Corrective vs. Palliative Surgery, *n* (%)	Corrective	63 (61)
	Palliative	41 (39)
Pulmonary Hypertension, *n* (%)		236 (40)
Baseline CRISP Score, Mean (SD)		4.13 (2.84)
Medication, *n* (%)	Peri-operative Inotropes	23 (3.9)
	Anti-failure Medication	397 (66)
	Beta Blocker	58 (9.7)
	Prostin Infusions	25 (4.2)
	Other Medication	123 (21)
Mortality		5 (0.8)

Overall, 137 (23%) patients experienced at least one complication (Table 2). Catheterisation related complications (CRC) were the most common 85 (14%), followed by anaesthetic related 48 (8%) and patient related complications 31 (5.2%). Most of the complications were moderate in severity, accounting for 95 (69.3%) cases. Major and catastrophic events were observed in 10 (7.5%) cases and 5 (3.6%) cases, respectively. In this cohort, all catastrophic events resulted in mortality. Minor complications occurred in 23 (16.8%) cases while 4 (2.9%) cases resulted in no physiological complications. The mortality rate was 0.8% (Table 1). Majority (60%) of the mortality was due to cardio-respiratory complications, while 40% of the mortality was from sepsis related complications (Supplementary Table S4). Most 417 (70%) patients were categorised into low- to intermediate risk categories (0–5 points), with 26 (4%) scoring ≥ 10 points. Bleeding was the most frequent catheter related complication, 47 (55.1%), whereas airway events predominated among anaesthetic related complications 27 (55%).

**Table 2:** Perioperative complications.

Characteristic *N* = 597		n (%)/Mean (SD)/Median (IQR)
Presence of Complication, *n* (%)	No Complication	460 (77)
	Any Complication	137 (23)
Categories of complications, *n* (%)	Patient Related	31 (5.2)
	Anaesthetic Related	48 (8.0)
	Catheterization Related	85 (14)
Severity of Complication, *n* (%)	None	4 (2.9)
	Minor	23 (16.8)
	Moderate	95 (69.3)
	Major	10 (7.4)
	Catastrophic	5 (3.6)
CRISP final score category, *n* (%)	0–2	163 (27)
	3–5	254 (43)
	6–9	154 (26)
	10–14	22 (3.5)
	15 or more	4 (0.5)
Patient Related Complication, *N* = 31	Pulmonary	11 (35.4%)
	Haemodynamic	17 (54,8%)
	Miscellaneous	3 (9.8%)
Catheterization Related Complication, *N* = 85	Vascular access related	47 (55.1%)
	Haemodynamic complications	22 (26%)
	Technical complications	14 (16.5%)
	Thrombus formation	2 (2.4%)
Anaesthetic Related Complication, *N* = 49	Airway management related	27 (55%)
	Ventilation related	13 (26,5%)
	Haemodynamic complications	9 (18,5%)

Patients who developed complications had significantly higher CRISP scores (median 6 vs. 3, *p* < 0.001). Complications were proportionally higher in children who were syndromic, had higher ASA class, were on perioperative inotropes, prostin, and beta blockers (Table 3). The proportion of complications increased progressively with higher CRISP categories. Univariable associations were found between complications and: higher CRISP score, higher ASA class, presence of syndromes, beta blocker therapy, and the use of perioperative inotropes. Patients younger than 1 year and with body weight under 5 kg had a univariable association with higher risk of complications.

**Table 3:** Relationship between demographic characteristics and complications.

Characteristic		No Complication *N* = 460	Any Complication *N* = 137	*p*-value
Age (years), Median (IQR)		2.0 (0.9–5.0)	1.0 (0.7–2.0)	<0.001
Age <1 year, *n* (%)		117 (25)	66 (48)	<0.001
Gender, *n* (%)	Female	217 (47)	67 (49)	0.722
	Male	243 (53)	70 (51)	
Weight (kg), Median (IQR)		10 (7–16)	7 (6–10)	<0.001
Weight <5kg, *n* (%)		30 (6.5)	26 (19)	<0.001
Haemoglobin (g/dL), Median (IQR)		12.5 (11.4–13.9)	12.3 (11.1–13.9)	0.243
Procedure Category, *n* (%)	Diagnostic	302 (66)	78 (57)	0.063
	Interventional	158 (34)	59 (43)	
Procedure Type, *n* (%)	PV Balloon valvuloplasty	8 (5.1)	10 (17)	0.026
	Other	35 (22)	15 (25)	
	PDA closure	106 (67)	33 (56)	
	VSD closure	9 (5.7)	1 (1.7)	
Elective vs. Emergency, *n* (%)	Elective	449 (98)	129 (94)	0.053
	Emergency	11 (2.4)	8 (5.8)	
ASA, *n* (%)	I	0 (0)	0 (0)	0.011
	II	83 (18)	19 (14)	
	III	351 (76)	101 (74)	
	IV	26 (5.7)	15 (11)	
	V	0 (0)	2 (1.5)	
Any Syndrome, *n* (%)		124 (27)	55 (40)	0.003
Syndromes, *n* (%)	Di George	30 (24)	19 (35)	0.390
	Down	82 (66)	32 (58)	
	Noonan	3 (2.4)	2 (3.6)	
	William	9 (7.3)	2 (3.6)	
Any Surgery, *n* (%)		85 (18)	19 (14)	0.212
Corrective vs. Palliative Surgery, *n* (%)	Corrective	52 (61)	11 (58)	0.791
	Palliative	33 (39)	8 (42)	
	No surgery	375 (82)	118 (86)	
Pulmonary Hypertension, *n* (%)		183 (40)	53 (39)	0.818
Medication, *n* (%)	Peri-operative Inotropes	4 (0.9)	19 (14)	<0.001
	Anti-failure Medication	307 (67)	90 (66)	0.820
	Beta Blocker	34 (7.4)	24 (18)	<0.001
	Prostin Infusions	15 (3.3)	10 (7.3)	0.038
	Other Medication	100 (22)	23 (17)	0.209
CRISP Score, Mean (SD)		3.69 (2.42)	5.59 (3.57)	<0.001
CRISP Category, *n* (%)	0–2	138 (30)	25 (19)	<0.001
	3–5	211 (46)	43 (32)	
	6–9	102 (22)	52 (39)	
	10–14	9 (2.0)	13 (7.0)	
	15 or more	0 (0)	4 (3.0)	
Cardiac Defect Subgroup, *n* (%)	Cono-truncal	7 (1.5)	2 (1.5)	0.195
	Cono-truncal (Complex)	9 (1.9)	5 (3.6)	
	Cono-truncal/Obstructive	48 (10.4)	19 (13.8)	
	Obstructive	34 (7.4)	16 (11.7)	
	Septal	136 (29.6)	32 (23.4)	
	Shunt	107 (23.3)	31 (22.6)	
	Complex	113 (24.6)	30 (21.9)	
	Miscellaneous	6 (1.3)	2 (1.5)	

In a multivariate regression analysis (Table 4), the CRISP score was significantly associated with complications (AOR 1.21 (95% CI 1.10–1.33; *p* < 0.001). Age under 1 year was associated with increased odds of complications (AOR 1.83 (95% CI 1.11–3.01; *p* = 0.018). The presence of any syndrome was independently associated with complications (AOR 1.72 95% CI 1.10–2.67; *p* = 0.016). Peri-operative inotropes (AOR 18.3 (95% CI 6.05–70.0; *p* < 0.001), beta blocker use (AOR 2.43 (95% CI 1.28–4.54; *p* = 0.006), and prostin infusions (AOR 0.25 (95% CI 0.07–0.80; *p* = 0.025) were independently associated with complications.

**Table 4:** Univariate and multivariate logistic regression models for any complication.

Characteristic		UOR (95% CI)	*p*-value	AOR (95% CI)	*p*-value
Age (years) *n*=597		0.87 (0.81 to 0.93)	<0.001		
Age <1 year *n*=597		2.73 (1.84 to 4.05)	<0.001	1.83 (1.11 to 3.01)	0.018
Gender *n*=597	Female	—			
	Male	0.93 (0.64 to 1.37)	0.72		
Weight (kg) *n*=597		0.95 (0.92 to 0.97)	<0.001		
Weight <5kg *n*=597		3.36 (1.90 to 5.91)	<0.001		
Haemoglobin (g/dL) *n*=597		0.98 (0.91 to 1.04)	0.49		
Procedure Category *n*=597	Diagnostic	—			
	Interventional	1.45 (0.98 to 2.13)	0.063		
Procedure Type *n*=217	PV valvuloplasty	—			
	Other	0.34 (0.11 to 1.03)	0.059		
	PDA closure	0.25 (0.09 to 0.68)	0.007		
	VSD closure	0.09 (0.00 to 0.62)	0.036		
Elective vs. Emergency *n*=597	Elective	—			
	Emergency	2.53 (0.96 to 6.39)	0.051		
ASA *n*=597	II	—			
	III	1.26 (0.74 to 2.22)	0.41		
	IV	2.52 (1.12 to 5.67)	0.025		
	V	9.253 (0.0 to NA)	0.98		
Any Syndrome *n*=597		1.82 (1.22 to 2.70)	0.003	1.72 (1.10 to 2.67)	0.016
Any Syndrome *n*=597	Di George	—			
	Down	0.62 (0.30 to 1.26)	0.18		
	Noonan	1.05 (0.13 to 6.92)	0.96		
	William	0.35 (0.05 to 1.55)	0.21		
Any Surgery *n*=597		0.71 (0.40 to 1.19)	0.21		
Surgery Type *n*=104	Corrective	—			
	Palliative	1.15 (0.41 to 3.13)	0.79		
Pulmonary Hypertension *n*=597		0.96 (0.64 to 1.41)	0.82		
Medication *n*=597	Peri-op Inotropes	18.4 (6.75 to 64.2)	<0.001	18.3 (6.05 to 70.0)	<0.001
	Anti-Failure	0.95 (0.64 to 1.43)	0.82		
	Beta Blocker	2.66 (1.50 to 4.65)	<0.001	2.43 (1.28 to 4.54)	0.006
	Prostin	2.34 (0.99 to 5.27)	0.044	0.25 (0.07 to 0.8)	0.025
	Other	0.73 (0.43 to 1.18)	0.21		
Previous Surgical Correction *n*=597		0.71 (0.40 to 1.19)	0.21		
CRISP Score *n*=597		1.26 (1.17 to 1.35)	<0.001	1.21 (1.10 to 1.33)	<0.001
CRISP Category *n*=597	0–2	—			
	3–5	1.12 (0.66 to 1.95)	0.67		
	6–9	2.81 (1.65 to 4.90)	<0.001		
	10–14	7.10 (2.43 to 21.6)	<0.001		
	15 or more	31,783 (0.0 to NA)	0.98		

## Discussion

The key findings of this study were that cardiac catheterisation was utilised for both diagnostic and interventional purposes, where majority of procedures were diagnostic and elective. There was a predominance of acyanotic lesions. Among cyanotic lesions, TOF was the most common. We observed a complication rate of 23%. Catheterisation related complications predominated, particularly vascular access-related complications which accounted for half of all complications. Majority of anaesthesia complications were airway management related, while majority of patient complications were haemodynamic in nature.

Cardiac catheterisation has advanced over the past twenty years, allowing minimally invasive procedures to increasingly replace or supplement surgery for certain conditions (16, 17). In line with the literature, the procedures were performed under general anaesthesia or deep sedation and required expertise in congenital cardiac physiology, non-operating room anaesthesia (NORA), and haemodynamic optimisation (16). Our patients similarly presented for catheterisation for corrective and palliation lesions, with syndromic comorbidities, and advanced disease, all of which increased peri-procedural risk (2, 18, 19). Anaesthetic considerations included lesion-specific physiology, the impact of anaesthetic agents on pulmonary and systemic vascular resistance, and the potential for sudden haemodynamic instability (13, 20).

The spectrum of congenital heart disease in this cohort was consistent with global literature (21). The prevalence of syndromes was also similar, with Down syndrome being the most frequently observed (22, 23). The disease profile highlighted the need for routine neonatal screening to detect congenital heart disease early in all children (2, 21, 24). Syndromes, including DiGeorge syndrome and Noonan syndrome, introduce perioperative risks such as immunodeficiency, bleeding diatheses, airway abnormalities, and altered physiological responses, which influence postoperative complications (25). Despite these complexities, the multidisciplinary perioperative care led to acceptable outcomes in this population.

Pulmonary hypertension was not associated with increased complications in this cohort. The higher prevalence thereof, when compared with rates typically reported in high-income countries, can be attributed to frequent delays in diagnosis and the timing of definitive cardiac surgery (19, 26). These factors are closely associated with increased morbidity and highlight the challenges faced in resource-limited settings. These findings continue to highlight disparities in morbidity and mortality rates between high-income countries (HICs) and low- and middle-income countries (LMICs) (1). Pulmonary arterial hypertension associated with CHD represented a significant contributor to morbidity and a potential influence on operability and outcomes (19, 26).

Two thirds of the cohort were on treatment for heart failure. This could also be attributed to delays in diagnosis and referral common in LMICs (21), particularly among children from rural or underserved regions without PCHD services (24, 27). As a result, many patients present with advanced disease and complications of uncorrected CHD, including cyanosis, congestive heart failure, arrythmias and pulmonary hypertension (13, 19). Aldersley et al., surveyed the availability and quality of PCHD services across Africa, where a total of 96 institutions across 45 countries responded (27). They found that only 18 countries (40%) offered complete PCHD services, meaning those countries could offer both cardiac catheterisation and cardiothoracic surgery with cardiopulmonary bypass. Ten countries (22%) provided cardiac surgery, but no interventional cardiac catheterisation and another 10 countries had no PCHD services at all.

There was an independent association between complications and a higher CRISP score, higher ASA class, presence of syndromes, beta blocker therapy, and the use of perioperative inotropes and prostin. The study found that patients younger than one year old, with body weight under 5 kg and those diagnosed with a syndrome faced a notably higher risk of complications during cardiac catheterisation. CRISP scores between 6 and 14 were significantly associated with complications. This indicates the major contribution of an immature or deranged haemodynamic and physiological state to a higher risk of severe adverse events (13, 15). Out of the 10 variables utilized to determine the CRISP score (15), our study confirmed both the statistical and clinical significance of the demographic and clinical presentation of the patients in our cohort, namely patient age, weight, haemodynamic stability (i.e., need for inotropes), organ dysfunction (e.g., heart failure), physiological parameters like the degree of cyanosis, severity of pulmonary hypertension and presence of dynamic right ventricular outflow tract obstruction (RVOT) (patients needing beta blockers) and complexity of congenital defects, i.e., duct dependant cardiac lesions needing prostin infusions. This supports the validity of the CRISP score as a robust risk stratification tool and its utility in predicting adverse events in paediatric cardiac catheterisation (28).

Preprocedural prostaglandin (Prostin) infusion was associated with a protective effect against adverse events. Prostaglandins are administered in neonates with duct-dependent critical congenital heart disease to maintain ductal patency and preserve pulmonary and systemic blood flow, preventing hypoxaemia and circulatory collapse (29). These patients typically present with features associated with higher CRISP scores and an increased procedural risk, including cyanosis, absent femoral pulses, need for emergency catheterisation, and ventilatory support. Despite this high-risk profile, patients receiving prostaglandin infusion (*n* = 25) demonstrated favourable outcomes, with no complications in 60% of cases, moderate complications in 32%, and only one major and one catastrophic complication. This may reflect early recognition of high-risk status, resulting in enhanced perioperative care, including senior multidisciplinary involvement, invasive monitoring, intensive care support, and expedited intervention. The observed protective effect is therefore more likely attributable to intensified clinical management and careful patient selection than to direct pharmacological effect of prostaglandins.

Similarly, the association between beta-blocker therapy and adverse events likely reflects greater underlying cardiac complexity [e.g., dynamic RVOT pathology, TOF, hypertrophic obstructive cardiomyopathy (HOCM), arrythmias etc.] and reduced haemodynamic reserve under anaesthesia, rather than a direct causal effect of the medication itself.

The identified risk factors for an increased risk of adverse events were similar to previous reports including younger age, lower body weight (16, 30), haemodynamic instability, extra-cardiac anomalies, complex cardiac lesions, emergency surgery and a higher ASA status (18). Data from the Paediatric Peri-Operative Cardiac Arrest (POCA) registry indicated that 30% of perioperative arrests occurred in patients with CHD in general, with 17% of these related to cardiac catheterisation (16, 18). The current study adverse events rate, compared to that reported by the international registry, Congenital Heart Disease Adjustment for Risk Method (CHARM), was three-fold higher 7.3% (13). The prevalence and type of complications were in keeping with literature (30, 31). The higher adverse events rate correlated with the higher risk profile of the patient profile, with similar findings in other LMICs (22).

Despite the complexity of the patient profile, the observed mortality in this cohort was notably low at 0.8%. This figure compares favourably compared with the reported mortality rate of 1.5% in a report from a low-income country facing similar challenges (22), although it remains approximately double the rate documented in HICs, where mortality rates range from 0.2% to 0.4% (17, 30). All the mortalities in our cohort occurred in patients with complex and critical congenital defects and were related to respiratory failure or cardiogenic shock in three patients (60%) and sepsis in two patients (40%).

Children with complex congenital heart disease are particularly vulnerable to infection due to repeated hospital admissions, malnutrition, chronic hypoxaemia, intensive care exposure and immunological vulnerability associated with syndromic disease (32). In addition, critically ill neonates and infants requiring prostaglandin infusions, perioperative inotropic support, or mechanical ventilation often experience prolonged hospitalisation, increasing their risk of nosocomial infection. Notably, the patients in our cohort who developed sepsis and septic shock acquired extracardiac infections, including meningitis and pneumonia, rather than complications directly attributable to cardiac catheterisation. These findings highlight the importance of comprehensive pre-procedural assessment, optimisation of concurrent infections, and careful patient selection and timing of cardiac catheterisation in high-risk patients.

Institutional experience and multidisciplinary expertise likely contributed to the relatively low mortality observed despite the high-risk patient profile (33). Although procedural volume classifications are difficult to define fairly in LMIC settings, the service functions as a major tertiary referral centre managing a substantial burden of complex congenital heart disease (34). The paediatric cardiology department is led by a senior cardiologist with over 30 years of experience who provides direct and indirect oversight of all cardiac catheterisation cases, likely contributing to consistent risk stratification, procedural planning and multidisciplinary perioperative management. These findings support the feasibility of achieving acceptable outcomes in resource-constrained environments when experienced specialist teams and structured multidisciplinary systems of care are established.

## Limitations

The weaknesses and limitations of the study include those expected in retrospective studies namely, 4.7% of missing data comprising 29 missing cardiac catheterisation reports and 1 illegible handwritten report. The study did not evaluate the impact of prematurity on the number of recorded patients with PDA, and this has the potential to distort spectrum of disease, procedure types and possibly AE rates. The study also did not look at the relationship between pulmonary hypertension and complications between age categories.

## Conclusion

The paediatric patients in our study show a similar spectrum of congenital cardiac defects, with higher burden of disease and a higher perioperative risk profile with those presented in LMICs. Despite this, severe adverse events were low, reflecting the feasibility and safety of providing complex cardiac interventions in a resource constrain health setting with an experienced and competent multidisciplinary team. Mortality was also very low. Risk stratification with tools such as CRISP score allowed for appropriate periprocedural planning, counselling of families and execution of care.

## Data Availability

The original contributions presented in the study are included in the article/Supplementary Material, further inquiries can be directed to the corresponding author.
