# Supplementary material for: Phenotypic profile and predictors of adverse outcomes during paediatric cardiac catheterisation at an academic hospital, South Africa
Source: Front Pediatr. 2026 Jun 25;14:1843252. doi: 10.3389/fped.2026.1843252 (PMC13346040; doi:10.3389/fped.2026.1843252)
Supplement: Supplementary file 1 [file Supplementaryfile1.docx]

**SUPPLEMENTARY MATERIAL**

**Supplementary Table 1:** [CRISP calculator](https://www.evidencio.com/models/show/678?v=1.19)

**Supplementary Table 2:** [**ccd26300-sup-0001-suppinfo.docx**](https://onlinelibrary.wiley.com/action/downloadSupplement?doi=10.1002%2Fccd.26300&file=ccd26300-sup-0001-suppinfo.docx)

**Supplementary Figure 1: Study Flow Diagram**

**Supplementary Table 3: Cardiac congenital lesions and categorization**

| **Congenital lesion classification** | **Cardiac lesion category, N = 597** | **Cardiac lesion type** | **n, (%)** |
| --- | --- | --- | --- |
| **Acyanotic, N=307 (51.4%)** | **Shunt, N= 139 (23.3%)** | Patent Ductus Arteriosus | 137 (23) |
|  |  | Patent Foramen Ovale | 1 (0.16) |
|  |  | Aorto-Pulmonary Window | 1 (0.16) |
|  | **Septal, N=168 (28.2%)** | Ventricular Septal Defect | 84 (14.1) |
|  |  | Atrio-Ventricular Septal Defect | 53 (8.9) |
|  |  | Atrial Septal Defect | 31 (5.2) |
| **Cyanotic, N=90 (15%)** | **Conotruncal, N=9 (1.5%)** | Double Outlet Right Ventricle | 5 (0.8) |
|  |  | Truncus Arteriosus | 4 (0.7) |
|  | **Conotruncal (obstructive), N= 67 (11.2%)** | Tetralogy Of Fallot | 53 (8.9) |
|  |  | Pulmonary Atresia | 14 (2.3) |
|  | **Conotruncal (complex), N= 14 (2.3%)** | Transposition Of Great Vessels | 14 (2.3) |
| **Obstructive, N=50 (8.4%)** | **Obstructive** | Coarctation of aorta | 17 (2.8) |
|  |  | Pulmonary stenosis | 16 (2.7) |
|  |  | Aortic stenosis | 12 (2.0) |
|  |  | Tricuspid atresia | 4 (0.7) |
|  |  | Ebstein’s anomaly | 1 (0.16) |
| **Complex, N=143 (24%)** | **Complex**  **All cardiac lesions with defects at more than one segment and or with a combination of defects.** | Complex | 17 (2.8) |
|  |  | Complex with septal defect | 47 (7.9) |
|  |  | Complex with shunt | 10 (1.7) |
|  |  | Complex with anomalous pulmonary circulation | 6 (1.0) |
|  |  | Complex with conotruncal | 42 (7.0) |
|  |  | Complex with obstructive | 20 (3.4) |
|  |  | Complex with hypoplastic left heart syndrome | 1 (0.16) |
| **Miscellaneous, N=7 (1.2%)** | **Anomalous pulmonary circulation, N= 3 (0.5%)** | Total Anomalous Pulmonary Venous Drainage | 1 (0.16) |
|  |  | Partial Anomalous Pulmonary Venous Drainage | 1 (0.16) |
|  |  | Major Aorto-Pulmonary Collateral Arteries | 1 (0.16) |
|  | **Anomalous systemic circulation, N= 3 (0.5%)** | Right aortic arch | 1 (0.16) |
|  |  | Aorto-atrial fistula | 1 (0.16) |
|  |  | Anomalous Left Coronary Artery from the Pulmonary Artery | 1 (0.16) |
|  | **Other, N=1 (0.16%)** | Cardiomyopathy | 1 (0.16) |

**Supplementary Table 4: Details of patients with mortality**

| Age (yrs) | Weight (kg) | Syndrome (yes / none) | Urgency of procedure | Cardiac diagnosis | Medication | Procedure type | CRISP score | Cause of death |
| --- | --- | --- | --- | --- | --- | --- | --- | --- |
| >1 yr | >5kg | Yes | elective | Complex | Antifailure | Diagnostic CC | 3 | Sepsis |
| >1yr | >5kg | None | elective | Conotruncal (obstructive) | Beta-blocker | Diagnostic CC | 6 | Hypercyanotic spells |
| 30 days – 1yr | 2.5 – 5kg | Yes | elective | Septal with pulmonary hypertension | Antifailure | Diagnostic CC | 8 | Respiratory failure |
| >1 yr | >5kg | None | elective | Complex with conotruncal (obstructive) | Beta-blocker | Diagnostic CC | 8 | Cardiopulmonary arrest |
| <30 days | <2.5kg | Yes | Emergency | Obstructive | Prostin infusion | Balloon valvuloplasty of PV | 11 | Sepsis |
